# Choledochal Cyst: A Retrospective Study of 30 Cases From Nepal

**DOI:** 10.7759/cureus.11414

**Published:** 2020-11-10

**Authors:** Narendra Pandit, Kunal Bikram Deo, Tek N Yadav, Sujan Gautam, Yogesh Dhakal, Lalijan Awale, Shailesh Adhikary

**Affiliations:** 1 Surgical Gastroenterology, B.P. Koirala Institute of Health Sciences, Dharan, NPL; 2 Anesthesiology and Critical Care, B.P. Koirala Institute of Health Sciences, Dharan, NPL

**Keywords:** choledochal cyst, hepaticojejunostomy, bile leak, malignancy, excision, nepal

## Abstract

Introduction

Choledochal cysts (CCs) are uncommon biliary lesions. Considering the evolution of imaging, we describe our experience with the presentation and management of choledochal cysts.

Methods

A review of the records of all patients with choledochal cyst managed in our institute were retrospectively analyzed. The study analyzed clinical presentation, diagnosis, treatment and postoperative outcomes.

Results

Between 2015 and 2019, 30 CCs (male/female: 7/23) were operated. We observed more adults compared to children (17 vs. 13). The median age at surgery was 18.5 years (4-67 years). The presentation included abdominal pain (90%), pancreatitis (17%0, cholangitis (13%), and incidental diagnosis in (7%). Anomalous union of the bile duct and the pancreatic duct was seen in 17%. Two patients had synchronous cholangiocarcinoma. The cysts were classified (Todani’s): I: 26; IV:3; and V: 1. The patients underwent complete excision of the cyst and Roux-en-Y hepaticojejunostomy - 27; pancreaticoduodenectomy - 1; hepaticoduodenostomy - 1; and cholecystectomy with T-tube drainage - 1 patient. The operative complications were observed in 10 (33.3%) patients: biliary leaks (four), superficial surgical site infections (four), and cholangitis (three). Only one patient developed a major complication; required re-operation for bile leak peritonitis. There was no operative mortality. One patient with cholangiocarcinoma died with the disease at three months of surgery. The remaining 29 patients are doing well at a mean follow-up of 29.5 months (12-56).

Conclusion

Adults CCs now far outnumber children at the time of presentation. The majority were symptomatic Todani’s type I cyst. Complete cyst excision and bilio-digestive anastomosis is the best treatment for type I and IV CCs, thus eliminating the risk of malignancy with an excellent operative outcome.

## Introduction

Choledochal cyst (CC) is a congenital condition of the biliary tract characterized by dilation of the extrahepatic and/or intrahepatic biliary tree. It has a female predominance (4:1), and its incidence varies with geographic location [1]. The entity is rare in the West, with the reported incidence being 1:200,000, while it is more common in Asian countries, with the incidence being 1:13,000 [2]. Most of the data and experience derives from Japan and Korea, where the entity is very common (1/1,000 population) [3,4]. It is usually caused by the anomalous pancreaticobiliary duct union, leading to the reflux of pancreatic juice into the biliary tree and subsequent biliary tree changes and dilatation [5,6]. Though the disease is congenital, it is frequently detected in early adulthood. The cyst can become complicated with stone disease, pancreatitis, cholangitis, and malignancy of the biliary tree. Hence, it needs to be treated by operative resection of the bile duct, cholecystectomy, and biliodigestive reconstruction, irrespective of the age at presentation [1,7].

There are several reports on outcomes of surgical treatment for CC in a large series of patients in other countries, but only anecdotal from Nepal. The study aims to present our experience in the management of CC in this retrospective review.

## Materials and methods

This is a retrospective observational study, which included all patients diagnosed and surgically treated for choledochal cyst at our academic institution (B.P. Koirala Institute of Health Sciences [BPKIHS]) from January 2015 to February 2019. The operative procedure was performed by the expert hepatobiliary surgical unit. The patients' medical records were reviewed to collect the following data: demographics and clinical information, imaging modality performed to diagnose/confirm, type of cyst as per Todani's classification, anomalous pancreaticobiliary duct union (APBDU), demonstrated by preoperative imaging or at the surgery and the operative details. The postoperative outcome measures, including complications, length of hospital stay, re-operation, and mortality were reviewed. Pathological reports of resected specimens were reviewed to confirm the presence of a choledochal cyst and any malignancy.

The follow-up visit was recorded for any late stricture or complications by clinical evaluation, liver function tests, and ultrasound. The study was approved by the Institute Ethics Committee and informed consent was taken from patients for the inclusion of their data for study purposes. Statistical analysis was performed with SPSS v. 17.0 software (SPSS Inc., Chicago, USA) for the descriptive statistical analysis by calculating the mean, median (range), and percentage where appropriate.

## Results

The choledochal cyst was reported in 32 patients during the study period, of which 30 patients were operated for it and records were available. Two patients refused surgery at the time of admission. The median age of the patients was 18.5 years (range: 4-67 years), with the predominance of female (n=23; 76.6%) population. The female to male ratio was 3.3:1. There were 13 (43.3%) pediatric (< 15 years) patients in the study. The adult CC patients were 56.7%. The main symptom was abdominal pain in 27 (90%) patients, jaundice in four (13.3%), pancreatitis in five (16.6%), and cholangitis in four (13.3%) patients. The classical triad of pain abdomen, jaundice, and the lump was seen in two (6.7%) - one each in the child and adult age group. Two children had an incidental diagnosis of CC at imaging.

**Figure 1:**
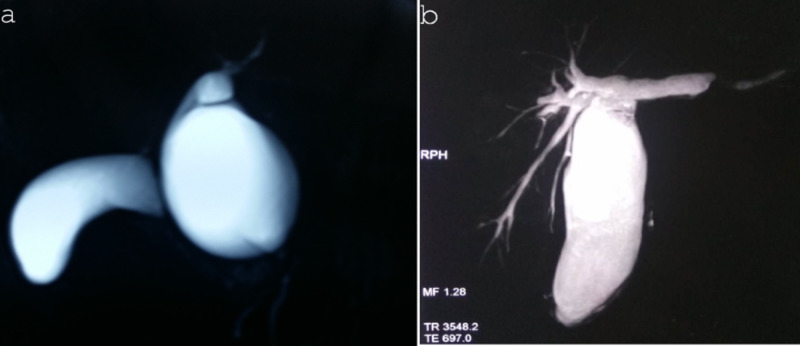
MRCP image showing choledochal cyst: (a) type Ia - cystic and (b) type Ic (fusiform) MRCP: magnetic resonance cholangiopancreatography

**Figure 2:**
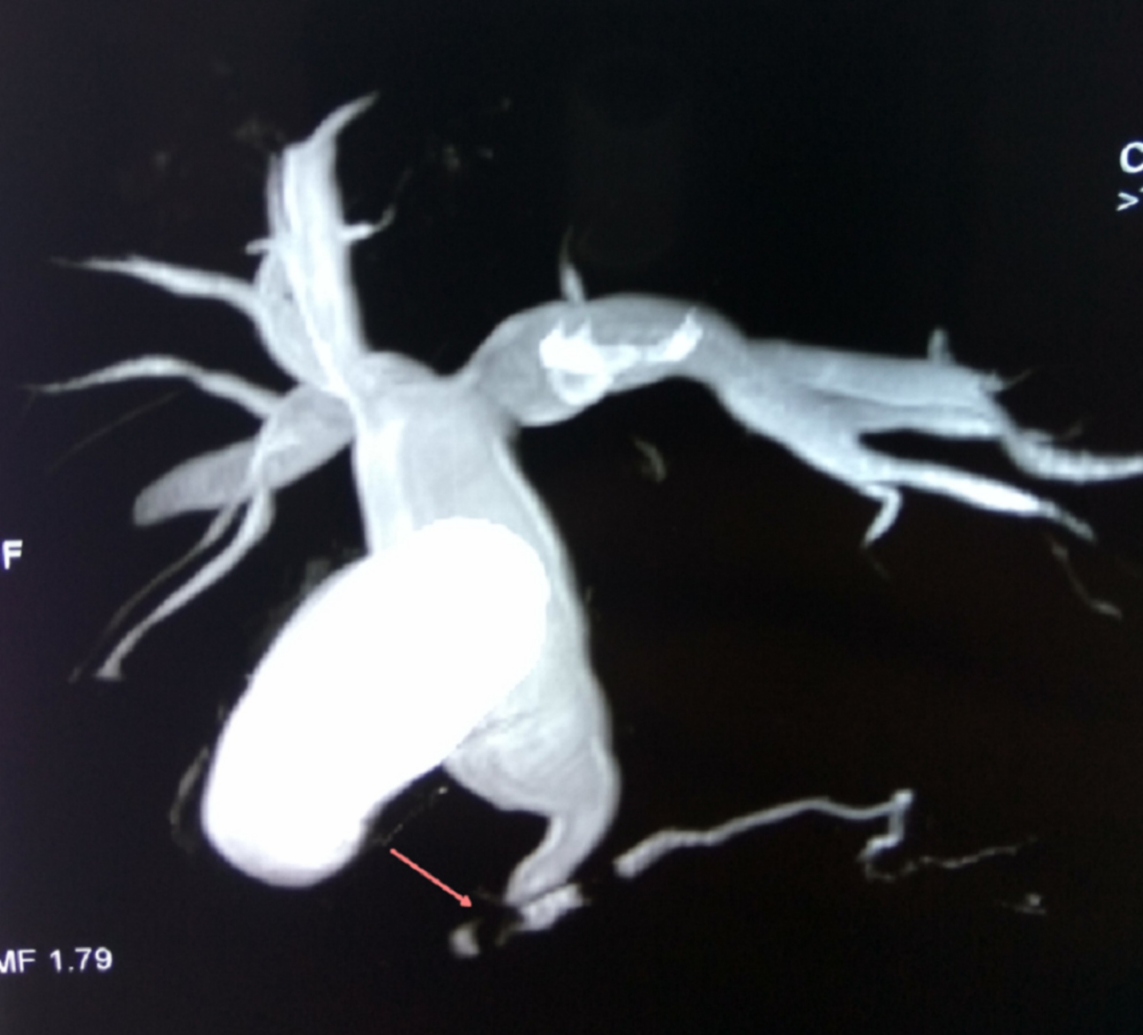
MRCP image showing type IV choledochal cyst with protein plug in the pancreatic duct and common channel (arrow) MRCP: magnetic resonance cholangiopancreatography

The diagnosis of the choledochal cyst was performed with ultrasound in all patients, and was later confirmed and surgically planned by magnetic resonance cholangiopancreatography (MRCP) in 14 (46.6%) and computed tomography in 10 (33.3%) patients. The most common type of choledochal cyst was a type I in 26 (86.6%), Type IV in 3 (10%), and type V in 1 (3.4%) (Figure 1 and Figure 2).

The APBDU was seen in five (16.6%) patients. All (30 patients) were treated with surgery. One patient of CC was previously misdiagnosed to have stone disease and underwent cholecystectomy and common bile duct exploration. Similarly, one patient required preoperative percutaneous transhepatic tube cholecystostomy for acute cholangitis with cholecystitis (Table 1).

**Table 1:** Demographics and clinical profile of patients (n=30) MRCP: magnetic resonance cholangiopancreatography; APBDU: anomalous pancreaticobiliary duct union

Parameters	n (%)
Age, median (years )(range)	18.5 (4-67)
Children (< 15 years)	13 (43.3%)
Female	23 (76.6%)
Duration of illness, median (months), range	5 (10 days to 60 months)
Presentation	
Pain abdomen	27 (90%)
Jaundice	4 (13.3%)
lump	4 (13.3%)
Preoperative evaluation	
Ultrasound	30 (100%)
Computed tomography (CT)	10 (33.3%)
MRCP	14 (46.6%)
Type of cyst (Todani's classification)	
Type I	26 (86.6%)
Type IV	3 (10%)
Type V	1 (3.4%)
Associated APBDU	5 (16.6%)
Associated pathology	
Cholangitis	4 (13.3%)
Cholelithiasis	7 (23.3%)
Hepatolithiasis	2 (6.7%)
Acute Pancreatitis	5 (16.6%)
Portal hypertension	2 (6.7%)
Chronic pancreatitis	1 (3.4%)
Prior biliary surgery	1 (3.4%)
Preoperative biliary drainage	1 (3.4%)

**Figure 3:**
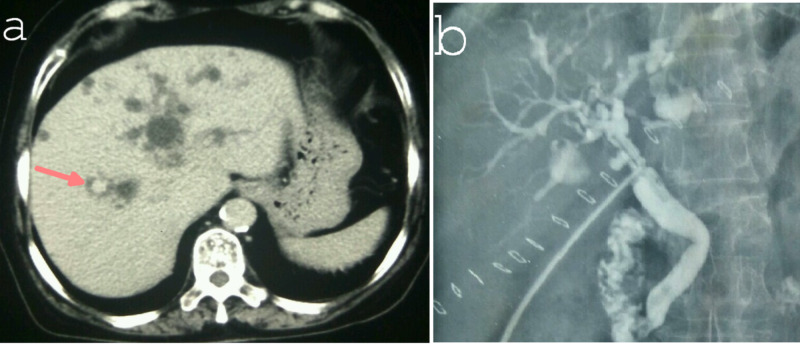
Contrast CT (a) and T-tube cholangiogram (b) showing type V choledochal cyst involving both the lobes of the liver. Note the characteristic “central dot sign” in the CT image of Caroli’s disease.

**Figure 4:**
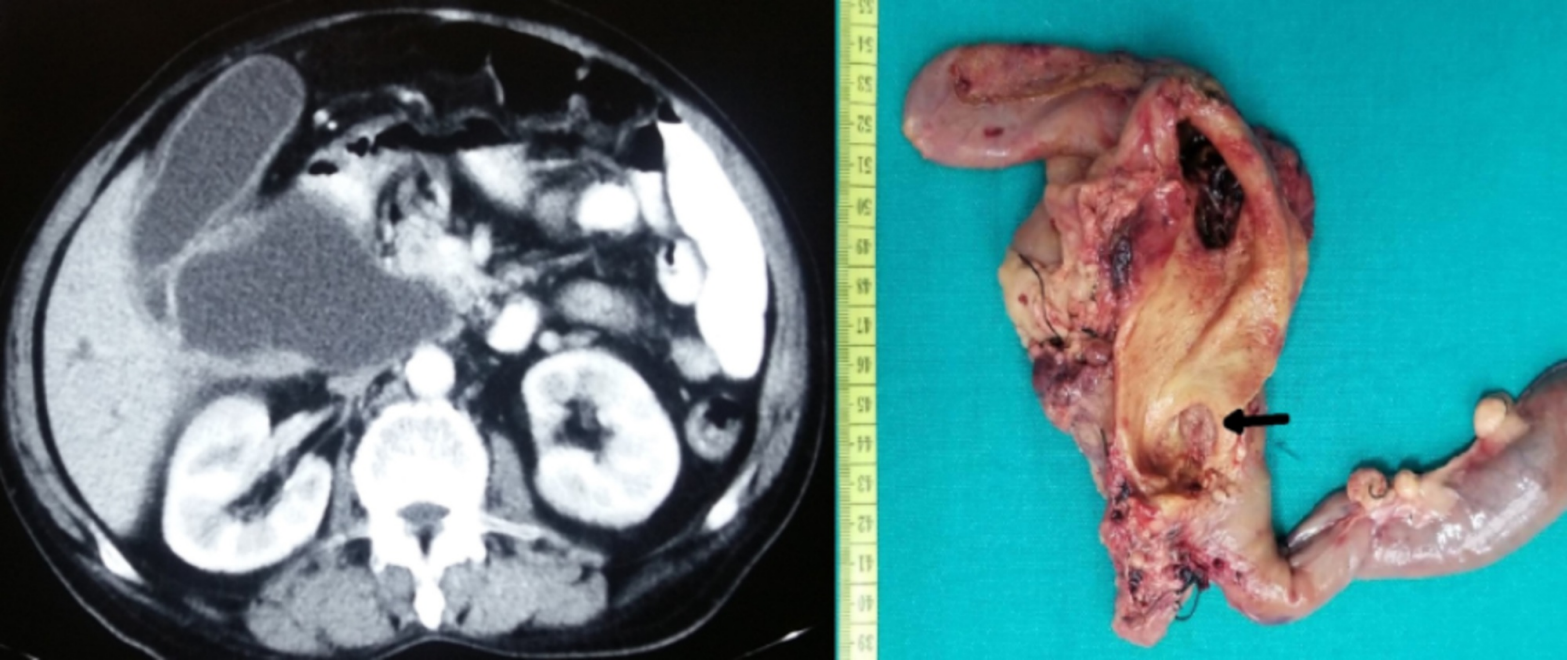
CT (cystic dilation of extrahepatic bile duct - 6 cm) and intraoperative image of pancreatoduodenectomy for choledochal cyst with malignancy (distal cholangiocarcinoma - arrow)

The operative procedures performed were: excision of cyst with Roux-en-Y hepaticojejunostomy (HJ) - 26 (86.6%); excision of a cyst, Roux-en-Y hepaticojejunostomy and lateral pancreaticojejunostomy for associated chronic calcific pancreatitis - 1 (3.4%); hepaticoduodenostomy (for metastatic disease) - 1 (3.4%); pancreaticoduodenectomy (distal cholangiocarcinoma) - 1 (3.4%); and cholecystectomy, T-tube drainage of bile duct for type V cyst (intraoperative hemodynamic instability) - 1 (3.4%) patient(s) (Figure 3 and Figure 4).

Postoperative complications were seen in 10 (33.3%) patients: bile leak - four (13.3%); superficial surgical site infection - four (13.3%), and cholangitis (managed conservatively) - three (10%) patients. All the complications were minor except, one seven-year-old child with bile leak (partial bilioenteric anastomotic dehiscence) and peritonitis required re-exploration, peritoneal lavage, and repair of HJ leak (over tube hepaticostomy - 10F infant feeding tube) on the second postoperative day. The child improved with it and got discharged on day 12. The external biliary fistula (from tube hepaticostomy) closed spontaneously on day 25. The patient with type V cyst, who underwent T-Tube drainage for hepatolithiasis, further underwent endoscopic sphincterotomy (Figure 3). Later, the T-tube was removed, and she is doing well at 36 months of follow-up.

The mean postoperative length of hospital stay was eight days (range: 4-15 days). There was no operative mortality. The histopathological report confirmed choledochal cyst in all patients. Two (6.7%) patients had concomitant cholangiocarcinoma (Figure 4). One diagnosed simultaneously in a 60-year-old male CC, underwent a pancreaticoduodenectomy (doing well at 12 months of follow-up), and the other 40-year-old female had metastatic disease at surgery, who simply underwent hepaticoduodenostomy and biopsy of the metastatic deposits. She died three months after surgery. The remaining 29 patients are doing well at a mean follow-up of 29.5 months (range: 12-56 months) (Table 2).

**Table 2:** Operative procedure and outcome of patients *Required re-exploration on the second day of surgery for HJ leak with biliary peritonitis HJ: Roux-en-Y hepaticojejunostomy; LPJ: lateral pancreaticojejunostomy; LOS: length of hospital stay; SSI: surgical site infection

Characteristics	N (%)
Type of surgery	
Excision + HJ	26 (86.6%)
Excision + HJ+ LPJ	1 (3.4%)
Hepaticoduodenostomy	1 (3.4%)
Pancreaticoduodenectomy	1 (3.4%)
Cholecystectomy +T-tube drainage	1 (3.4%)
Overall operative morbidity	10 (33.3%)
SSI	4 (13.3%)
Bile leak	4 (13.3%)
Cholangitis	3 (10%)
Re-operation*	1 (3.4%)
Malignancy	2 (6.7%)
Postoperative LOS (days), mean (range)	8 (4-15)
Operative mortality	none
Follow-up, months (mean)(range)	29.5 (12-56)
Re-stricture (HJ site)	none

## Discussion

This series of patients with choledochal cyst disease represents the largest Nepalese experience. Our series consists predominantly of adults (57%) and females (76%). Though the disease is classically regarded as a condition of children, it was more often observed in adult females signifying the shift in presentation from the first decade of life to adulthood. Additionally, four of our patients with CC were in their sixth decade of life at the presentation. We observed far more symptomatic adults with complications (n=23; 76%) in our cohorts. The predominant CCs were type I (86%). The classical triad of jaundice, abdominal pain, and right upper quadrant lump was observed in only 6.7% of patients.

The basic pathophysiology mechanism for CC includes APBDU, which is seen in 30% to 70% of all CC; more frequent in children compared to adults [3,8]. This APBDU results in complications like cholangitis, pancreatitis, portal hypertension, liver function tests derangement, and malignancy [2]. In the Indian series (n=132), it was observed in 28% of patients [9]. Similarly, in the Western (n=394) and the Eastern series, it was detected in 12% and 90% of patients respectively [10-12]. In our cohort, it was observed in only 16% of patients, which is in line with other data from different countries. The low APBDU rate may be secondary to the few patients (47%) undergoing MRCP in our series, under-efforts, and inexperience by the radiologists on the multi-planar image to detect it, lack of “long common channel” of the bile duct and pancreatic duct and lack of real-time cholangiography to diagnose ineffective duodenal sphincter due to APBDU.

The current standard of care of type I and IV CC is complete excision of the cyst with bilio-digestive reconstruction by Roux-en-Y HJ. The extent of cyst excision includes hilum proximally (till the non-dilated biliary tree) up to the pancreaticobiliary junction distally (funneling of the dilated bile duct) [2,13,14]. The cyst excision extent is aided by the assistance of opening of the cyst in the mid-portion coupled with intraoperative choledochoscopy as was performed by the authors in the present series [15]. Type V cyst (Caroli’s disease) treatment ranges from resection of the disease if unilobar/segmental involvement to liver transplantation for the diffuse disease [8]. When non-feasible due to the comorbidity or disease condition, a conservative treatment approach like endoscopic sphincterotomy prevents recurrent episodes of cholangitis due to biliary sludge/debris and further liver damage as was performed in one of our patients [11]. Similarly, CC with bile duct stone disease may be under-treated with cholecystectomy with bile duct exploration (one patient in the present series). Hence, there should be a high index of suspicion of CC in patients with dilated common duct (> 1.5 cm), without intrahepatic bile duct dilatation.

The overall morbidity in the surgical treatment of CC ranges between 13% to 40% [3,16]. The majority being the wound-related or bilioenteric anastomotic leak. Bile leak is also the predominant cause of mortality [1,9,10]. In the present series, the operative complication was seen in 33% of the patients; 4 (13%) being bile leak, while one patient required reoperation for major bile leak and peritonitis. Postoperative cholangitis was observed in 10%, which is in agreement with the published reports (8%-10%) [9,17]. The risk factor for bile leak is childhood CC, as the cyst is thin-walled and membranous. Hence, the anastomosis should be performed meticulously, with fine interrupted absorbable suture (5-0). If reoperation is required, percutaneous tube hepaticostomy from the distal jejunal limb helps divert the bile externally and facilitates healing as was performed in our series. Similarly, none developed delayed anastomotic stricture or malignancy at a mean follow-up of 29 months in our series.

The incidence of bile duct malignancy in patients with CC is 20 times higher than the general population. One of the Japanese studies reported 16% of patients with CC or APBDU had concomitant cancer [18]. In a recent meta-analysis of the risk of malignancy in CC disease; the risk was 11% with the median age at presentation being 36 years [19]. The largest western experience from John Hopkins reported 5.4% (5/92 patients) malignant disease present at the time of resection of CC [1]. In our study, it was observed in 2 (6.7%) patients and matches with other international studies. Contrarily, a recent Japanese study (n=110) had no biliary cancer in their series at the time of definite operation; however, the age at surgery in the series was 12 days to 17 years [20]. No doubt the risk of malignancy increases with each decade of life; and risk persists even after cyst excision (0.75%-5.4%) [19]. Malignancy risk is 1% at 10 years, 15% at 20 years, 26% at 40 years, and 45% at 70 years [2]. Hence, biliary cancer is rare if excision is performed in less than 10 years of age.

The present study has some limitations. It was a single-center retrospective study with a relatively small number of patients. However, this is the first report from Nepal to evaluate the clinical profile and the results of surgical treatment with a 100% minimum follow-up of one year.

## Conclusions

In conclusion, reviewing our institutional experience of 30 patients with CC, we found that adults outnumber children at the time of presentation. The majority were type I cyst and symptomatic often with complications. Synchronous malignancy of the biliary tract was observed in 6.6% of patients. Complete excision of the cyst and Roux-en-Y hepaticojejunostomy is the best treatment for type I and IV CCs, thus eliminating the symptoms and risk of malignancy with an excellent operative outcome. The surgery should be performed by the expert hepatobiliary surgeon for the best outcome.
